# Fetal Brain Infection Is Not a Unique Characteristic of Brazilian Zika Viruses

**DOI:** 10.3390/v10100541

**Published:** 2018-10-03

**Authors:** Yin Xiang Setoh, Nias Y. Peng, Eri Nakayama, Alberto A. Amarilla, Natalie A. Prow, Andreas Suhrbier, Alexander A. Khromykh

**Affiliations:** 1 Australian Infectious Diseases Research Centre, School of Chemistry and Molecular Biosciences, The University of Queensland, St. Lucia 4072, Australia; y.setoh@uq.edu.au (Y.X.S.); y.g.peng@uq.net.au (N.Y.P.); a.amarillaortiz@uq.edu.au (A.A.A.); 2 Inflammation Biology Group, QIMR Berghofer Medical Research Institute, Brisbane 4006, Australia; Eri.Nakayama@qimrberghofer.edu.au (E.N.); Natalie.Prow@qimrberghofer.edu.au (N.A.P.); 3 Department of Virology I, National Institute of Infectious Diseases, Tokyo 162-8640, Japan

**Keywords:** Zika virus, pregnancy, fetal infection, congenital Zika syndrome, Asian lineage

## Abstract

The recent emergence of Zika virus (ZIKV) in Brazil was associated with an increased number of fetal brain infections that resulted in a spectrum of congenital neurological complications known as congenital Zika syndrome (CZS). Herein, we generated *de novo* from sequence data an early Asian lineage ZIKV isolate (ZIKV-MY; Malaysia, 1966) not associated with microcephaly and compared the *in vitro* replication kinetics and fetal brain infection in interferon α/β receptor 1 knockout (IFNAR1^−/−^) dams of this isolate and of a Brazilian isolate (ZIKV-Natal; Natal, 2015) unequivocally associated with microcephaly. The replication efficiencies of ZIKV-MY and ZIKV-Natal in A549 and Vero cells were similar, while ZIKV-MY replicated more efficiently in wild-type (WT) and IFNAR^−/−^ mouse embryonic fibroblasts. Viremias in IFNAR1^−/−^ dams were similar after infection with ZIKV-MY or ZIKV-Natal, and importantly, infection of fetal brains was also not significantly different. Thus, fetal brain infection does not appear to be a unique feature of Brazilian ZIKV isolates.

## 1. Introduction

Zika virus (ZIKV) belongs to the genus *Flavivirus* of the Flaviviridae family [1], which includes yellow fever virus, dengue virus, West Nile virus, Japanese encephalitis virus, and tick-borne encephalitis virus. First isolated in Uganda in 1947, ZIKV has since spread across continents, emerging as a medically significant pathogen associated with Guillain–Barré syndrome in adults and, following infection of pregnant women, with a spectrum of congenital neurological complications known as congenital Zika syndrome (CZS) that manifests in the newborns [2]. ZIKV is an enveloped virus with a single-stranded positive-strand RNA genome of approximately 11 kb in length. Like all flaviviruses, the ZIKV genome encodes a single open reading frame translated into a single polyprotein that is cleaved post-translationally by cellular and viral proteases into three structural proteins, i.e., capsid (C), precursor membrane (prM), envelope (E), and seven non-structural (NS) proteins (NS1, NS2A, NS2B, NS3, NS4A, NS4B, NS5) [3].

Phylogenetically, ZIKV strains are categorized into either African or Asian lineages [4], with most epidemic-associated ZIKV strains belonging to the Asian lineage [4,5,6,7,8]. Several epidemiological and bioinformatics studies investigating the etiology of ZIKV emergence in the Americas have independently suggested that amino acid changes within the Asian lineage viruses were likely to have contributed to enhanced infectivity and pathogenicity, which resulted in the unprecedented increase in CZS that was primarily associated with the Brazilian outbreak [8,9,10,11,12]. The ability to infect a developing fetus and cause CZS, including microcephaly [13,14], is the most distressing feature of ZIKV. In the 2015 Brazilian outbreak, microcephaly in newborns was reported to be 20 times higher than the background incidence [15], with a retrospective analysis of the 2013 outbreak in French Polynesia also showing an increased risk of microcephaly associated with ZIKV infection [16].

A central question associated with the ZIKV epidemic in Brazil (and in French Polynesia) is whether the virus strains involved had acquired mutations that enhanced their ability to cause CZS [17,18]. Several studies have supported this notion [19,20], whereas others have argued that all ZIKV strains may be similarly neurotropic [21,22].

We previously generated the contemporary Asian lineage isolate ZIKV-Natal from sequence data obtained directly from the brain tissue of an aborted ZIKV-infected human fetus during the 2015 Brazilian outbreak [14,23]. Using a pregnant IFNAR1^−/−^ mouse model, we previously demonstrated that the Brazilian isolate (ZIKV-Natal) was capable of causing fetal infection and congenital malformations [23,24]. Herein, we sought to determine whether fetal infection is a newly acquired characteristic of Asian lineage strains of ZIKV by comparing ZIKV-Natal to the P6-740 Malaysian 1966 ZIKV isolate (ZIKV-MY). ZIKV-MY was chosen because it represents the earliest documented Asian lineage ZIKV strain, with no reported association with human congenital disease [25]. We show that ZIKV-MY and ZIKV-Natal produced similar viremias in pregnant interferon α/β receptor 1 knockout (IFNAR1^−/−^) dams and were both capable of causing fetal brain infections.

## 2. Materials and Methods

### 2.1. Generation of ZIKV-MY and Chimeric Virus Using Circular Polymerase Extension Reaction (CPER)

**Figure 1:**
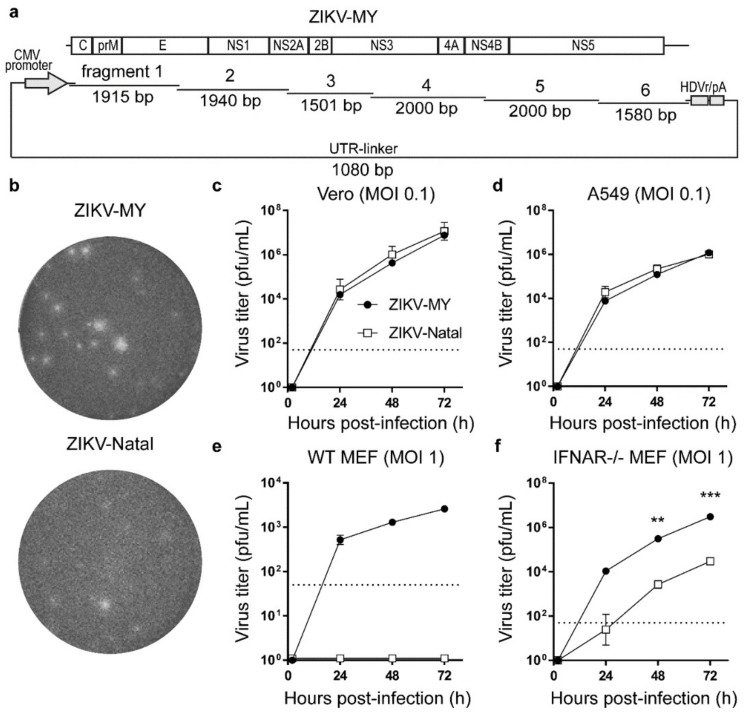
De novo generation and characterization of ZIKV-MY. (**a**) Schematic of Circular Polymerase Extension Reaction (CPER) fragments used for recovering ZIKV-MY. All fragments, except for the UTR-linker, are drawn to scale; (**b**) Plaque morphology on a Vero cell monolayer of ZIKV-MY recovered from the culture supernatant of CPER-transfected Vero cells, compared to ZIKV-Natal; Growth kinetics of ZIKV-MY versus ZIKV-Natal was performed on (**c**) Vero, (**d**) A549, (**e**) WT MEF, and (**f**) IFNAR^−/−^ MEF cells at their indicated multiplicity of infection (MOI), and culture supernatants were harvested at the indicated time points post-infection and titered by plaque assay. The dashed lines represent the limit of detection of the assay. Means and ± SE are shown. Statistical analyses were performed using *t*-tests (*n* = three biological replicates); statistically significant are differences shown in panel (**f**) **—*p* = 0.008, ***—*p* < 0.001.

The ZIKV-MY (strain P6-740) isolate whose sequence was used in this study was reported to be passaged six times in suckling mouse brains, once in baby hamster kidney (BHK) cells, once in C6/36 cells, twice in Vero cells, and five times in Vero E6 cells (GenBank accession number KX694533). Six dsDNA fragments covering the entire viral sequence (Figure 1a) were purchased from Integrated DNA Technologies (Baulkham Hills, NSW, Australia) as gBlocks, and each cloned into a pUC19 vector to produce six plasmids. The plasmids were sequenced to confirm the correct viral sequence. The fragments used for CPER (see Figure 1a) were then amplified from individual pUC19 plasmids using corresponding pairs of primers (Table 1). CPER assembly and recovery of WT ZIKV-MY virus by transfection into Vero cells were performed as previously described [23]. The WT ZIKV-Natal virus was generated previously [23]. The ZIKV-Natal/MY-prME chimeric virus was generated using CPER by replacing the ZIKV-Natal fragments 1 and part of fragment 2 encompassing E gene (Figure 1a) with those of ZIKV-MY. Chimeric primers (Table 1) were used for polymerase chain reaction (PCR) amplification of amplicons to generate chimeric virus.

### 2.2. Growth Kinetics

Vero (CCL-81), A549 (ATCC CCL-185), wild-type mouse embryonic fibroblasts (MEFs), and IFNAR^−/−^ MEFs (44) were infected with passage 1 of C6/36-derived stock of ZIKV-MY, ZIKV-Natal, or ZIKV-Natal/MY-prME viruses at the indicated multiplicity of infection (MOI), and 200 µL of culture supernatant was collected from each sample well at the indicated times post-infection. Three independent experiments were conducted for each cell line. Viral titers were determined by standard plaque assay on Vero cells, as previously described [23].

### 2.3. Mice and ZIKV Infection

IFNAR1^−/−^ mice on a C57BL/6J background were provided by Dr P Hertzog (Monash University, VIC, Australia) [26] and bred in-house at the Queensland Institute of Medical Research (QIMR) Berghofer Medical Research Institute. Pregnant female IFNAR1^−/−^ mice (between 10–20 weeks old) were infected s.c. at the base of the tail with 100 µL of medium at a dose of 10^4^ CCID_50_ as described [24]. Mating was verified by the presence of a vaginal plug. Pregnancy was monitored by weight gain. In older (>10 weeks) IFNAR1^−/−^ mice, infection with either ZIKA-MY or ZIKV-Natal did not result in any overt morbidity. Mice were euthanized with CO_2_.

### 2.4. Ethics Statement

All mouse work was conducted in accordance with the “Australian code for the care and use of animals for scientific purposes”, as defined by the National Health and Medical Research Council of Australia. Animal experiments and associated statistical treatments were reviewed and approved by the QIMR Berghofer Medical Research Institute animal ethics committee (P2195, A1604-611M).

### 2.5. ZIKV CCID_50_ Assays

ZIKV cell culture infectious dose, 50% endpoint (CCID_50_) assays for viremia and tissue titers were undertaken as described [27,28], with minor modifications. Briefly, serum or supernatants from tissues (bead-macerated in medium) were collected and titrated in quadruplicates in five-fold serial dilutions on low-passage C6/36 cells (ATCC CRL-1660). After five days, 25 µL of the supernatants were individually transferred onto parallel plates (i.e., A1 to A1, A2 to A2… H12 to H12, etc.) containing Vero E6 cells (ATCC CRL­1586). After seven more days, the plates were stained with crystal violet to visualize cytopathic effects (CPE). The titers were calculated using the Reed and Münch method.

### 2.6. Statistics

Statistical analysis of experimental data was performed using IBM SPSS Statistics for Windows, Version 19.0. Two-sample comparison using t-test was performed when the difference in variances was <4, skewness was >−2, and kurtosis was <2. Non-parametric data with difference in variances of <4 was analyzed using Mann–Whitney U-test; if the difference of variances was >4, the Kolmogorov–Smirnov test was employed.

## 3. Results

### 3.1. De Novo Generation and Characterization of ZIKV-MY

To generate ZIKV-MY, six synthetic gBlock DNA fragments covering the full genome of ZIKV-MY (GenBank: KX694533) were purchased from Integrated DNA Technologies (Figure 1a). The first 28 nucleotides of the 5′-UTR were missing from the published sequence, and, therefore, a fully conserved sequence of this region from four complete genomes of Asian lineage ZIKV (AGTTGTTGATCTGTGTGAATCAGACTGC, GenBank: KU527068, KU501215, KU681081, KU681082) was used instead. The gBlock fragments were individually cloned into pUC19 plasmids for archival purposes and then re-amplified for assembly by CPER, with the addition of the UTR-linker fragment, as described previously [23]. The CPER DNA was transfected into Vero cells, the culture media collected at six days post-transfection, and the presence of the virus was confirmed by plaque assay method as previously described [23] (Figure 1b).

Analysis of replication efficiencies in different cell lines showed that ZIKV-MY and ZIKV-Natal replicated with similar efficiencies in Vero and A549 cells (Figure 1c,d). As reported previously [23], ZIKV-Natal was unable to replicate in wild-type (WT) mouse embryonic fibroblasts (MEFs), while, here, we show that ZIKV-MY replicated to a detectable, albeit still relatively low, level (~2.57 × 10^3^ pfu/mL) (Figure 1e). Even in MEFs deficient in the type I IFN response (interferon α/β receptor-deficient MEF; IFNAR^−/−^ MEF), ZIKV-MY replicated more efficiently than ZIKV-Natal (Figure 1f). To ascertain whether the structural proteins of ZIKV-MY contributed to improved replication in murine cells, a chimeric virus with ZIKV-MY prME proteins and ZIKV-Natal non-structural proteins was constructed (ZIKV-Natal/MY-prME) (Figure S1a). In both WT and IFNAR^−/−^ MEFs, the chimeric virus expressing ZIKV-MY prME did not show enhanced replication compared to ZIKV-Natal (Figure S1b,c). The better replication of ZIKV-MY in MEFs is clearly unrelated to the type I IFN response and would appear to be imparted by non-structural proteins.

### 3.2. ZIKV-MY and ZIKV-Natal Both Infect IFNAR1^−/−^ Mice and Produce Similar Viraemia and Viral Titres in Different Organs

**Figure 2:**
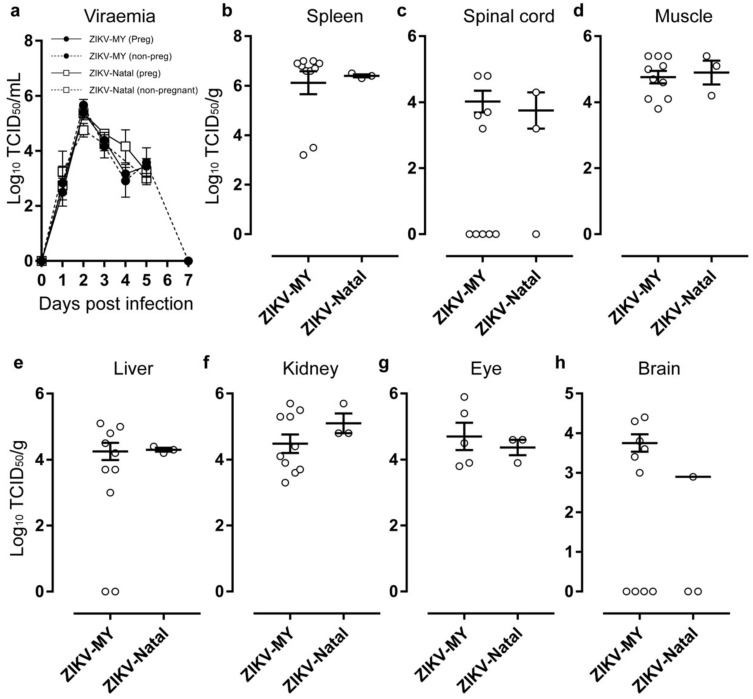
ZIKV-MY and ZIKV-Natal replicate to similar levels *in vivo.* (**a**) Interferon α/β receptor 1 knockout (IFNAR1^−/−^) C57BL/6 female mice were infected with ZIKV-MY or ZIKV-Natal. Pregnant mice were infected at E12.5 of gestation. Viremia was determined daily for five days post-infection. Pregnant mice were euthanized at E17.5, and their (**b**) spleen, (**c**) spinal cord, (**d**) muscle, (**e**) liver, (**f**) kidney, (**g**) eye, and (**h**) brain samples were processed to determine the viral titers by the CCID_50_ assay.

IFNAR1^−/−^ mice were infected with ZIKV-MY or ZIKV-Natal at E12.5 of gestation, and viremias were determined daily for five days post-infection. No statistically significant differences in mean viremias were observed after infection with the two viruses in either pregnant or non-pregnant mice (Figure 2a). Viremia in the non-pregnant mice were measured up to day 7 post-infection and showed that the virus was cleared by 7 days post-infection (Figure 2a). The pregnant mice were euthanized on day 5 post-infection (E17.5), and a panel of organs was harvested (spleens, spinal cords, muscles, liver, kidneys, eyes, brains) to determine the viral titres. No statistically significant differences in viral titres for any organs were observed between ZIKV-MY and ZIKV-Natal infections (Figure 2b–h).

### 3.3. ZIKV-MY and ZIKV-Natal Infection of Placenta and Foetal Heads in Pregnant IFNAR1^−/−^ Mice

**Figure 3:**
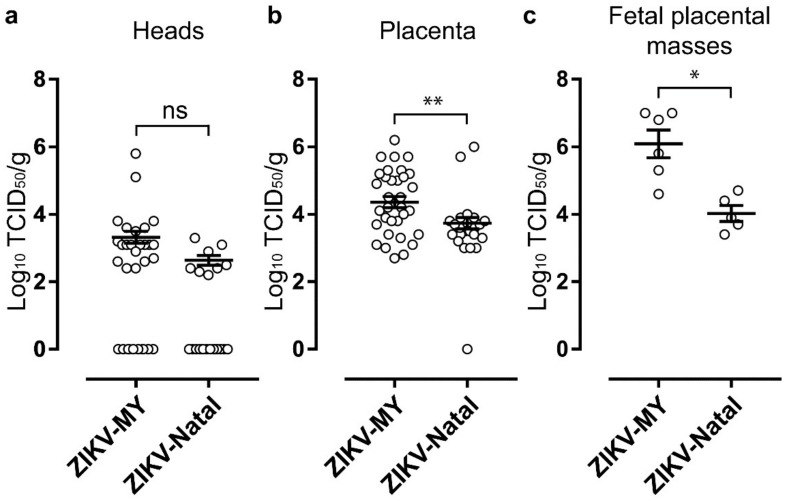
Fetal and placental virus titers after infection with ZIKV-MY or ZIKV-Natal. IFNAR1^−/−^ C57BL/6 pregnant mice were infected with ZIKV-MY or ZIKV-Natal at E12.5 and were euthanized at E17.5 of gestation. (**a**) Fetal heads, (**b**) placenta, and (**c**) deformed fetal–placental masses were collected, and tissue viral titers determined by the CCID_50_ assay. *—*p* = 0.002; **—*p* = 0.045.

Groups of pregnant mice were infected with ZIKV-MY or ZIKV-Natal at E12.5 of gestation and euthanized at E17.5, with fetuses and placentas collected. The viral tissue titres in fetal heads obtained from dams infected with ZIKV-MY or ZIKV-Natal were not significantly different (Figure 3a, *p* = 0.077 Kolmogorov–Smirnov test). Placental titers were about 0.8 logs higher for ZIKV-MY than for ZIKV-Natal infection (*p* = 0.002, Kolmogorov–Smirnov test). Following ZIKV infection, placenta and fetuses often form highly deformed indistinguishable masses, as described previously [23]. Viral titers in these masses were ≈2 logs higher (*p* = 0.045, Kolmogorov–Smirnov test) in ZIKV-MY- than in ZIKV-Natal-infected dams (Figure 3c). These data provide no support for the notion that ZIKV-Natal (unequivocally associated with microcephaly) has acquired elevated capacity to infect fetal brains.

## 4. Discussion

Herein, a 1966 Malaysian isolate of ZIKV was generated de novo directly from a published sequence and was shown to cause fetal brain infection in pregnant IFNAR1^−/−^ mice, with similar efficiency to the 2015 Natal isolate from Brazil. Both ZIKV-MY and ZIKV-Natal also generated indistinguishable viremia and viral loads in a panel of organs tested. Thus, the propensity for congenital infection does not appear to be a unique feature of contemporary Brazilian ZIKV strains.

The use of mice as a ZIKV animal model remains the main practical approach for *in vivo* characterization of ZIKV virulence. Because of the inability of ZIKV to degrade murine STAT2 [29], IFN response-deficient mice are required, and previously we have validated the use of IFNAR1^−/−^ C57BL/6 as a congenital infection model [23]. In other studies that investigated fetal infection by ZIKV in a mouse model [18,30,31,32,33,34], direct comparisons between pre-outbreak versus outbreak ZIKV strains were not performed. Although one study [35] did compare the pre-outbreak 1966 Malaysian strain to an outbreak Puerto Rico 2015 strain of Asian lineage, viral pathogenesis was examined only in the context of an infection in non-pregnant adult mice, rather than investigating fetal infection in pregnant dams. Here, we have directly compared the infection of pregnant dams and their fetuses after subcutaneous infection with Malaysian and Brazilian isolates and show that both strains are capable of infecting fetal brains.

A potential limitation of our study is that the Malaysian isolate generated herein was derived from the sequence of an isolate that had undergone six passages in suckling mouse brain (Table 2). Potentially, such passaging may have resulted in the selection of a virus with increased ability to mediate congenital infections in mouse models. However, on the basis of the amino acid alignment, only two amino acids in ZIKV-MY (NS4B I180 and NS5 Y720) are found in one of two other mouse-adapted isolates (Table 2), and the rest of the amino acid changes are either found in non-mouse-adapted isolates or not found in any other isolates (Table 2). NS4B I180 and NS5 Y720 are also unlikely to be associated with congenital ZIKV syndrome [9].

In conclusion, our results argue that ZIKV has an intrinsic ability to cause congenital infection, irrespective of the sequence evolution that has been documented in recent years.

## Figures and Tables

**Table 1:** Primers used for generation of cDNA amplicons of parental P6-740 Malaysian 1966 Zika virus isolate (ZIKV-MY) and ZIKV-Natal (Brazilian isolate)/MY chimeric virus.

WT ZIKV-MY Primers	Primer Sequence (5′ to 3′)
Fragment 1 Forward	AGTTGTTGATCTGTGTGAATCAGAC
Fragment 1 Reverse	GTGAACGCCGCGGTACATAAGGAGTATG
Fragment 2 Forward	CATACTCCTTATGTACCGCGGCGTTCAC
Fragment 2 Reverse	GATGAAAGAGACCAGCAACGCGGG
Fragment 3 Forward	CCCGCGTTGCTGGTCTCTTTCATC
Fragment 3 Reverse	CAGCAGCGACAACCCTGGTTGGAG
Fragment 4 Forward	CTCCAACCAGGGTTGTCGCTGCTG
Fragment 4 Reverse	GTACATGTAGTGCGCCACGAGCAGAATG
Fragment 5 Forward	CATTCTGCTCGTGGCGCACTACATGTAC
Fragment 5 Reverse	CTCCTGGTGTGCGGCTCATTTCTTC
Fragment 6 Forward	GAAGAAATGAGCCGCACACCAGGAG
Fragment 6 Reverse	AGACCCATGGATTTCCCCACACCG
Chimeric primers	Primer sequence (5′ to 3′)
Natal 5′C (MY1966) R	CTCCCACGTCTGGTGACCTCCACTGCCATAGCTGTGGTCAGCAG
MY1966prM_F	GTGGAGGTCACCAGACGTG
MY1966E_R	AGCAGAGACGGCTGTAGATAGG
Natal NS1-junc2 (MY1966) F	CTTCCTATCTACAGCCGTCTCTGCTGATGTGGGGTGCTCGGTG

**Table 2:** Amino acid differences between ZIKV-MY and ZIKV-Natal.

Protein	Amino Acid Position (Within Protein)	African, MR766 1947 (146× SM, 1× C6/36, 1× Vero)	African, Nigeria 1968 (21× SM, 1× Vero)	Malaysian ZIKV-MY 1966 (6× SM, 1× BHK, 1× C6/36, 8× Vero)	African, DakAr41525 1985 (5× Vero, 1× C6/36, 2× Vero)	Brazilian ZIKV-Natal 2015 (No Passaging)	Brazilian, Suriname 2015 (4× Vero)
prM	1	A	A	V	A	A	A
	17	S	S	S	S	N	N
	21	K	K	K	K	E	E
	31	V	V	V	V	M	M
E	154–156	NDT	Deleted	NDI	NDT	NDT	NDT
	393	D	D	D	D	E	E
	473	V	V	V	V	M	M
	487	T	T	T	T	M	M
NS1	146	E	K	K	K	E	K
	188	V	V	A	V	V	V
	233	T	T	T	T	A	T
	264	V	V	V	V	M	M
	349	M	M	M	M	V	M
NS2A	117	A	A	V	A	A	A
	143	A	A	A	A	V	V
NS3	400	N	N	N	N	H	H
	472	M	M	M	M	L	L
	584	Y	Y	Y	Y	H	H
NS4B	14	A	A	G	A	S	S
	26	M	V	M	M	I	I
	49	L	L	L	L	F	F
	98	M	M	M	M	I	I
	180	I	A	I	V	V	V
	184	V	V	V	V	I	I
	186	L	L	L	L	S	S
	240	T	T	T	T	I	T
NS5	115	M	M	T	M	V	V
	140	P	P	S	P	P	P
	230	I	I	I	I	T	T
	268	A	A	A	A	V	V
	276	M	M	L	M	M	M
	283	I	I	V	I	I	I
	377	N	N	N	N	S	S
	527	A	A	T	A	I	I
	531	K	K	K	K	R	R
	588	G	G	R	G	K	K
	643	P	P	P	P	S	S
	648	R	R	S	R	N	N
	704	S	S	S	S	D	D
	714	H	H	Y	H	H	H
	720	Y	H	Y	H	H	H
	868	D	D	D	D	N	N

Accession numbers: ZIKV-MY (KX694533), ZIKV-Natal (KU527068), MR766 (HQ234498), Nigeria (HQ234500) DakAr41525 (KU955591), Suriname (KU312312). SM—suckling mouse brain.

## Supplementary Materials

The following are available online at http://www.mdpi.com/1999-4915/10/10/541/s1, Figure S1: Chimeric ZIKV encoding prM-E genes from ZIKV-MY and the remaining genome of ZIKV-Natal.
