# Supplementary material for: Fetal Brain Infection Is Not a Unique Characteristic of Brazilian Zika Viruses
Source: Viruses. 2018 Oct 3;10(10):541. doi: 10.3390/v10100541 (PMC6213914; doi:10.3390/v10100541)
Supplement: Supplementary file 1 [file viruses-10-00541-s001.pdf]

Supplementary Materials:

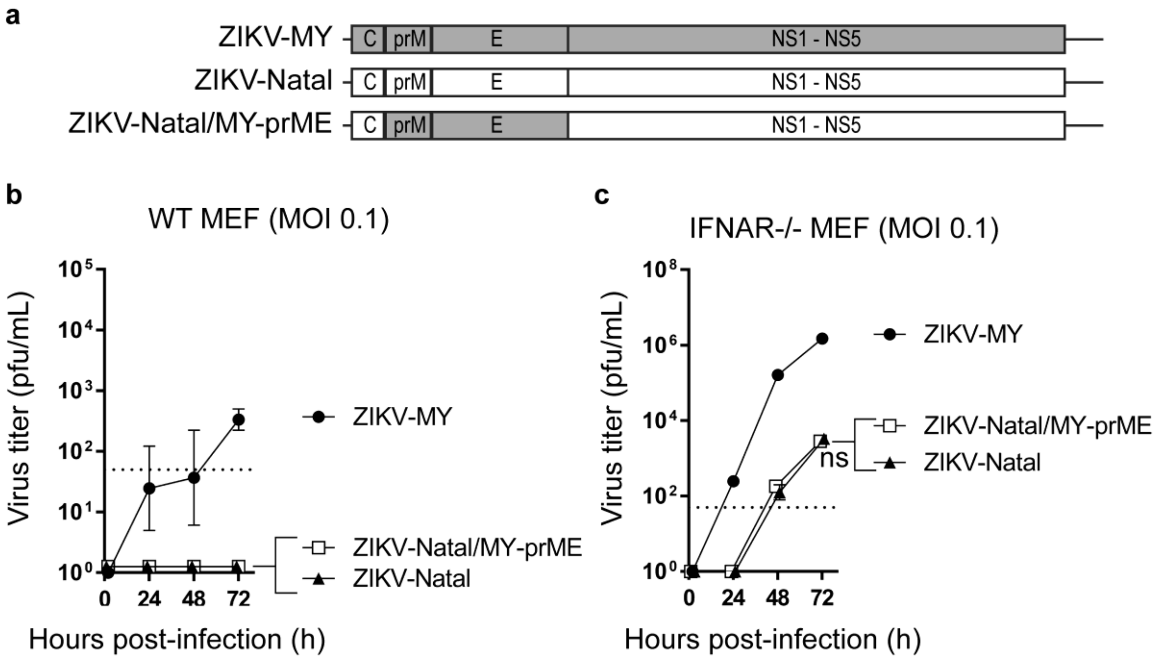

Figure S1. Construction and characterization of ZIKV-Natal/MY-prM/E chimeric virus. (**a**) Schematic depicting the ZIKV-MY and ZIKV-Natal parental viruses, and the constructed ZIKV-Natal/MY-prME chimeric virus; Growth kinetics comparing ZIKV-MY, ZIKV-Natal, and ZIKV-Natal/MY-prME was performed on (**b**) WT and (**c**) IFNAR$^{-/-}$ MEF cells at an MOI of 0.1, and culture supernatants harvested at the indicated time points post infection and titered on Vero cell by plaque assay.
